# Ion-Exchange Chromatography Coupled With Dynamic Coating Capillary Electrophoresis for Simultaneous Determination of Tropomyosin and Arginine Kinase in Shellfish

**DOI:** 10.3389/fchem.2018.00305

**Published:** 2018-07-25

**Authors:** Linglin Fu, Jinru Zhou, Chong Wang, Xiaohui Li, Lei Zheng, Yanbo Wang

**Affiliations:** ^1^Food Safety Key Laboratory of Zhejiang Province, School of Food Science and Biotechnology, Zhejiang Gongshang University, Hangzhou, China; ^2^Zhejiang Engineering Institute of Food Quality and Safety, Zhejiang Gongshang University, Hangzhou, China; ^3^School of Food Science and Engineering, Hefei University of Technology, Hefei, China

**Keywords:** capillary electrophoresis, tropomyosin, arginine kinase, allergen, shellfish, food safety, ion-exchange chromatography

## Abstract

Tropomyosin (TM) and arginine kinase (AK) are known as two major allergens in seafood. For the first time, we demonstrate a newly developed ion-exchange chromatography coupled with dynamic coating capillary electrophoresis (IEC-DCCE) method to simultaneously analyze the TM and AK in shellfish. First, we have optimized the procedure of IEC for simple enrichment of TM and AK crude extract. By using 30 mM borate-borax at pH 9.0 with 0.3% (v/v) Tween-20 as a dynamic coating modifier for capillary electrophoresis (CE) separation, the migration time, separation efficiency and electrophoretic resolution greatly improved. The limits of detection (LOD) were 1.2 μg mL^−1^ for AK and 1.1 μg mL^−1^ for TM (S/N = 3), and the limits of quantification (LOQ) were 4.0 μg mL^−1^ for AK and 3.7 μg mL^−1^ for TM (S/N = 10). The recovery of AK ranged from 91.5 to 106.1%, while that of TM ranged from 94.0 to 109.5%. We also found that only when the concentrations of AK and TM were above LOD reported here, these proteins can stimulate human mast cell (LAD2) degranulation. Finally, the use of IEC-DCCE to analyze fresh shellfish samples highlights the applicability of this method for the simultaneous detection of these allergens in complex food systems.

## Introduction

Due to the rich nutrients and savory flavor, seafood has become a staple of human diets worldwide. Unfortunately, fish and shellfish, two of the “big eight” categories of food allergens, are responsible for almost 90% of food allergies (Fernandes et al., 2015). It is generally considered that shellfish and fish most commonly provoke severe food anaphylaxis, while the prevalence of a shellfish allergy is usually higher than that of a fish allergy. A USA-based study demonstrated a prevalence of fish allergy of ≤ 0.2% of children and ≤ 0.5% of adults and a shellfish allergy in ≤ 0.5% of children and ≤ 2.5% of adults (Gray et al., 2015). In Asian countries, recent data highlight seafood as a significant sensitizer in up to 40% of children and 33% of adults (Lopata and Lehrer, 2009).

Currently, with the development of aquatic product processing technology, the possibility that indistinguishable seafood allergenic ingredients in some intensively processed food products makes allergic individuals at higher risk of coming in contact with allergenic foods. Hence, the accurate detection and labeling of allergen information on food packages is of great significance in avoiding harmful allergen contact and food safety (Reese et al., 2015). In accordance with the European Union (EU) regulations (Directive 2003/89/EC), the mandatory labeling and highlighting of 14 allergens, including the very potentially risky group, shellfish allergens, has been declared (Taylor and Baumert, 2015). Therefore, it is urgently required to develop reliable, highly sensitive and feasible analytical methods for detecting and labeling allergenic ingredients in food products in order to help industry and government management of seafood allergens. In addition, evaluating the efficiency of allergen elimination approaches also requires effective detection methods.

Importantly, development of effective detection methods relies on the identification of various allergens in given food raw materials, such as all kinds of shellfish. Until now, a number of allergens have been identified in shellfish, among which tropomyosin (TM) and arginine kinase (AK) are considered the two major allergens responsible for most shellfish-caused allergic reactions and even cross-react with insect-derived ones (Shafique et al., 2012). TM in muscle cells was first described as a heat-stable IgE-binding shellfish allergen in shrimp (Hoffman and Day, 1981). Due to the high sequence homology and structural similarity of TM in different shellfish species, the total serum IgE from almost 75% of the individuals who are allergic to TM in one shellfish species can cross-react with that in other shellfish species (Tsabouri et al., 2012). AK, a phosphagen kinase, is involved in cell metabolism of invertebrates and was first reported as an allergen in the Pacific white shrimp species (Lit v 2) (Garcíaorozco et al., 2007) and further described in other crustaceans with high sequence identity compared to Lit v 2 (Yadzir et al., 2012).

So far, there are two kinds of food allergen analytical methods that are widely applied: protein-based methods, including enzyme-linked immunosorbent assays (ELISAs) and liquid chromatography (LC)-coupled mass spectrometry (MS), and DNA-based methods, mostly referring to all kinds of polymerase chain reaction (PCR) methods (van Hengel, 2007). ELISA, a commercially available method, detects food allergens by utilizing antibodies for target allergenic proteins. It has been reported that two sandwich ELISA methods were established by using specific monoclonal antibodies (mAbs) to quantify tropomyosin in seafood raw materials (Zhang et al., 2014) and commercial shrimp, crab, and lobster extracts (Jeoung et al., 1997). In the case of DNA-based methods, real-time PCR has been applied to analyze tropomyosin in blue crab (Cal s 2) and tiger prawn (Pen m 1) (Eischeid et al., 2013). However, ELISA is prone to cross-reactivity of antibodies with non-target food components leading to false-positive results. DNA-based methods depend on the sequence of the target gene such that they are not suitable for quantifying gene-unidentified target allergenic proteins in food. Although Carrera et al. have developed a LC-MS/MS approach to select 19 parvalbumin (an allergen in fish) peptide biomarkers in 16 species of fish, application of LC-MS in seafood allergen detection still requires further study and validation (Carrera et al., 2012).

Alternatively, capillary electrophoresis (CE), which shows obvious advantages of simplicity of operation, high speed, and low sample and reagent consumption, is considered a practical and powerful tool for detecting multiple large and small molecules simultaneously (Guo et al., 2015; Sille and Kašička, 2016). CE has been successfully applied to the analysis of some kinds of allergens, such as birch pollen allergen (Bet v 1a) (Punzet et al., 2006), allergenic extracts from olive pollen (Zienkiewicz et al., 2014), camel milk proteins (β-lactoglobulin, α-lactalbumin, lactoferrin and serum albumin) (Omar et al., 2016), wheat proteins (Piergiovanni, 2016), and cow milk allergens (Gasilova et al., 2014). However, so far, there is no report on the application of CE in the analysis of seafood allergens, including major allergens such as TM and AK.

In terms of biochemical characteristics of TM and AK, they have very close molecular weights and isoelectric points, which makes it difficult to distinguish them with general CE. In addition, the variety of large background proteins from shellfish samples also becomes a bottleneck in analyzing TM and AK by CE without any modifications. Ion-exchange chromatography (IEC) was a simple and effective analytical tool for the fractionation of crude protein mixtures by salt-gradient (Schmidt et al., 2014). Therefore, the current study explores the first use of a newly developed ion-exchange chromatography coupled with dynamic coating capillary electrophoresis (IEC-DCCE) platform to simultaneously analyze the two main allergens (TM and AK) of shellfish. Dynamic coating was used to improve repeatability, resolution, and operability by reducing electroosmotic mobility (EOF) and minimizing the adsorption of analytes on the capillary wall. Furthermore, we evaluated our strategy in fresh shellfish samples by determination of sensitivity, reproducibility and resolution, and the allergenicity of TM and AK could be observed only when their concentration was above the limit of detection (LOD) of our approach.

## Experimental

### Human blood samples

Serum samples of 30 subjects with confirmed clinical history of allergic responses to *Penaeus chinenis* were obtained as a normal routine procedure during the allergic disease diagnostic workup from the second affiliated hospital of Zhejiang University School of Medicine, Hangzhou, Zhejiang, China. Aliquots of these stored at −80°C until further use. Informed consent was obtained from each volunteer. Oral informed consent was obtained from all participants before enrolment in the study. This procedure as well as the whole study were done in accordance with good clinical practice guidelines and approved by Zhejiang Gongshang University Ethics Review Committee and Ethics Committee of Zhejiang University as it is part of a routine procedure where no additional consent is required by law.

### Chemicals and materials

Hydroxymethyl aminomethane (Tris), polyethylene oxide (PEO), dodecyl sodium sulfate (SDS), boric acid, Tween-20 and sodium chloride were purchased from Aladdin, Los Angeles, Southern California. All chemicals used were of analytical grade. DEAE-Sepharose fast flow was purchased from General Electric Company, Fairfield, Connecticut. Positive blood serum from patients allergic to shellfish was kindly offered by the second affiliated hospital of Zhejiang University School of Medicine, Hangzhou, Zhejiang, China. LAD2 cells were obtained from ATCC (Rockefeller, Maryland). Tropomyosin (TM) and arginine kinase (AK) were obtained from GenScript, Piscataway, New Jersey. A total of 10 species of worldwide high-consumption of shellfish (*Litopenaeus vannamei, Fenneropenaeus chinensis, Trachypenaeus curvirostris, Penaeus monodon, Macrobrachium nipponense, Procambarus clarkia, Metapenaeus ensis, Exopalaemon carinicauda, Eriocheir sinensis, Portunus trituberculatus*) were purchased from a local supermarket (CenturyMart, Hangzhou, China). All solutions were prepared using ultrapure water (18.2 MΩ) purified using a Milli-Q system (Millipore, Billerica, Massachusetts).

### Sample preparation and ion-exchange chromatography

Shrimp muscle was ground into powder with liquid nitrogen. Shrimp powder (0.1 g) was weighed into a 1.5-mL centrifuge tube, and then, 1 mL of mixture buffer (1 mL of Lysis Buffer, 1 μL of protease inhibitor, 10 μL of phosphodiesterases, 5 μL of 100 mM PMSF) was added and mixed well at 4°C. Total protein extraction was performed using a commercial kit (KeyGEN, Nanjing, China), and protein extract was obtained after stationary incubation for 2 h at 4°C. Then, the extract was centrifuged in a Microfuge 22 R Centrifuge (Beckman Coulter, Mississauga, ON) at 10,000 × *g* at 4°C for 5 min. The supernatant was eluted with 20 mL of NaCl (0.3 M) at 1 mL min^−1^ in Tris-HCl buffer (pH 7.5) using DEAE-Sepharose Fast Flow column. The concentration of total or column-collected proteins was determined by bicinchoninic acid (BCA) assay (Pierce, Rockford, USA) with bovine serum albumin (BSA) as the standard. The purity of TM and AK in crude protein extracts and effluents was analyzed by sodium dodecyl sulfate-polyacrylamide gel electrophoresis (SDS-PAGE) using AlphaView SA 3.4.0 software (Proteinsimple, California, USA).

### Capillary electrophoresis analysis

**Figure 1:**
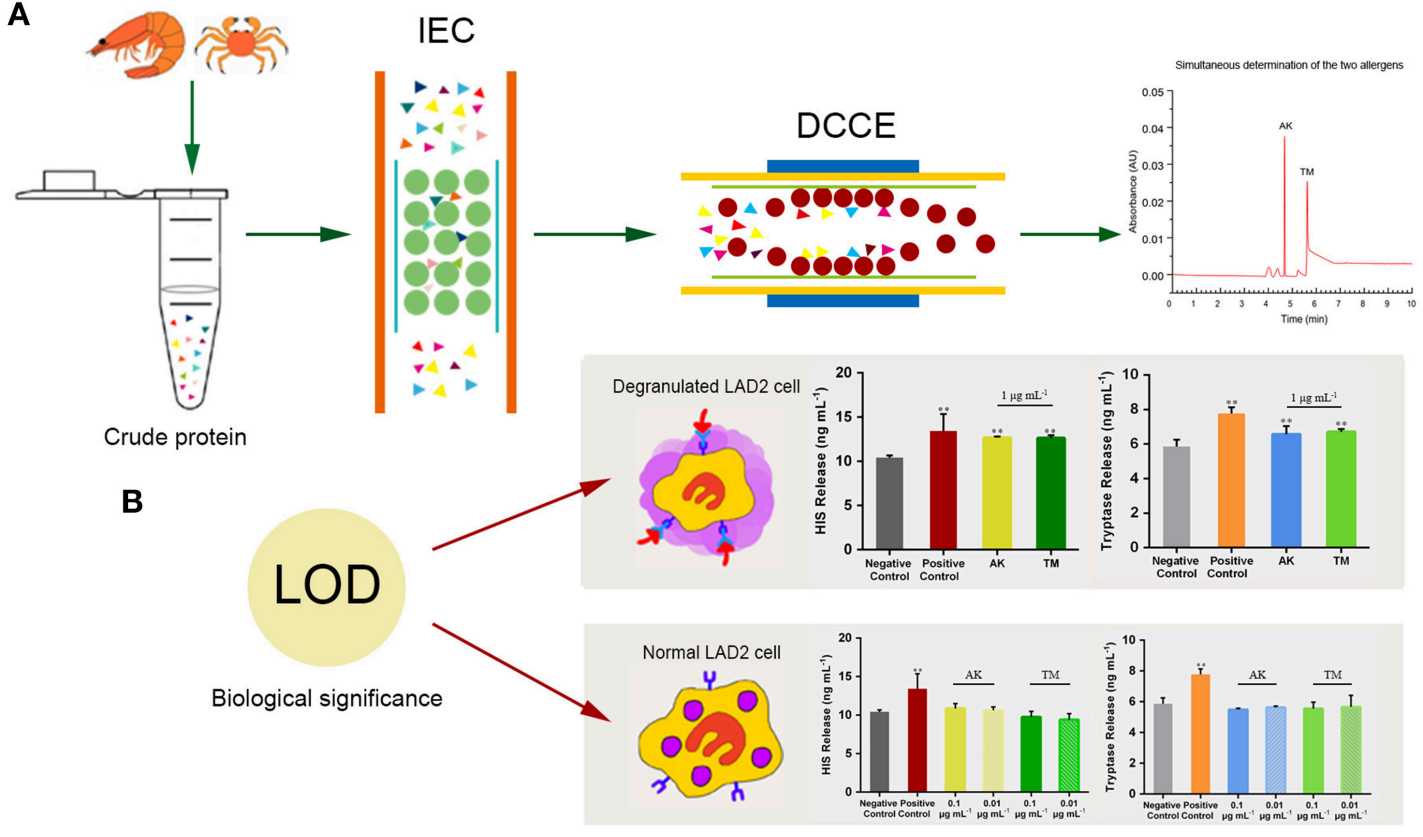
**(A)** Schematic illustration of the process of allergen detection and **(B)** biological significance of the LOD. **p < 0.001 compared with negative control.

The capillary zone electrophoresis (CZE) analysis was performed on a Beckman P/ACE MDQ CZE system with UV detector (214 nm). A 32-Karat software was used for controlling the instrument. Fused silica capillaries of 75 μm i.d., 375 μm o.d. (20 cm effective length and 30.2 cm total length) were obtained from Beckman Coulter, Mississauga, ON. For all experiments, 0.3% Tween-20 was added as a dynamic coating agent into 100 mL of 30 mM sodium borate, which was finally selected as the running buffer. The pH of the running buffer was adjusted to 9.0 with the addition of 0.1 g boric acid powder, and then the solution was filtered (0.45 μm) and ultrasonically degassed for 20 min. The sample was injected under 0.5 psi for 5 s and run on 18 kV with a positive high voltage. Figure 1 illustrates the method for recognizing and quantifying the target allergens and biological significance of the LOD of IEC-DCCE. In shellfish sample analysis, the external standard method was used for quantification of AK and TM. Standard curves were made with different concentrations (5~50 μg mL^−1^) of AK and TM and analyzed in triplicate.

### Cell culture and degranulation

LAD2 human mast cells (Kirshenbaum et al., 2003) were cultured in serum-free RPMI 1640 medium (GIBCO, Los Angeles, southern California) supplemented with 9% (v/v) fetal bovine serum and 1% (v/v) penicillin-streptomycin. Cells (1 × 106 cells/mL) were incubated in a 24-well plate with 10 μL of shellfish allergic-IgE antibody sera (2 ng mL^−1^) in triplicate for 2 h at 37°C. After centrifugation at 900 × g for 5 min at 4°C, cell pellets were washed and resuspended in 4-(2-hydroxyethyl)-1-piperazineethanesulfonic acid (HEPES) buffer [10 mM HEPES, 137 mM NaCl, 2.7 mM KCl, 0.38 mM Na_2_HPO_4_·7H_2_O, 5.6 mM glucose, 1.8 mM CaCl_2_·H_2_O, 1.3 mM MgSO_4_·7H_2_O, 0.4% bovine serum albumin (BSA), pH 7.4]. Then, the cell suspensions were stimulated with three concentration gradients (1, 0.1, and 0.01 μg mL^−1^) of AK and TM for 30 min at 37°C. Meanwhile, the cells were supplemented with keyhole limpet hemocyanin (KLH) as a negative control and compound 48/80 (C48/80) as a positive control (Pundir et al., 2014). After centrifugation at 900 × g for 5 min at 4°C, the supernatants were collected for ELISA, and the cells were treated for transmission electron microscopy.

### Transmission electron microscopy

LAD2 cells were fixed with glutaraldehyde fixative (2.5% glutaraldehyde in 0.05 M cacodylate buffer with 0.1 M NaCl, pH 7.5) at room temperature for 20 min. Sections were cut with a knife on a LEICA EM UC7 ultratome (LEICA, Solms, Hessen) and observed using a Hitachi H-7650 transmission electron microscope (TEM) (Hitachi, Tokyo, Japan).

### ELISA analysis

A double-antibody sandwich ELISA was performed to determine the histamine levels in the cell supernatants using a Human Histamine (His) ELISA Kit (BIM, San Francisco, CA). Tryptase in the cell supernatants was assayed using a Human Tryptase (MCT) ELISA Kit (BIM, San Francisco, CA) according to the manufacturer's instructions.

### Statistical analysis

Statistical significance of the data was determined by one-way analysis of variance using SPSS.16.0 (IBM SPSS, Armonk, NY, USA). A *p* < 0.05 was considered statistically significant.

## Results and discussion

### Optimization of sample preparation

As the aim was to develop a platform for simultaneously analyzing TM and AK in shellfish samples using the potential of the CZE separation and quantification method suitable for quick detection in practical applications, we optimized the procedure of enrichment of TM and AK crude extract. First, total proteins were obtained with 21 fractions including AK and TM (Figure S1). The purity of TM was ~9.11% as analyzed by Alpha View SA 3.4.0, while that of AK was ~11.37%. Second, ion-exchange chromatography (IEC) was performed to remove non-targeted proteins and reduce possible interference during CZE separation and quantification. The column was washed by running a linear saline gradient buffer (0.1–0.5 M NaCl), and three major absorption peaks were present during the flows (Figure S2). Specifically, AK with a molecular weight of 40 kDa appeared in peak 1 eluted by 0.1 M NaCl, while the 36 kDa TM had 0.3 M NaCl in the peak 3 (Figure S2). On the basis of the above results, we ultimately employed 0.3 M NaCl as the elution buffer for IEC. Our findings showed that the preparation of TM (14.56%) and AK (17.48%) IEC treated protein extract achieved a cleaner background with many fewer other protein bands compared to the crude protein extract (Figure S1). This procedure of sample treatment mainly dependent on IEC provides the foundation for further characterization of TM and AK by CE simultaneously and accurately at practically feasible separation efficiency.

### Optimization and dynamic coating selection of CZE separation conditions

**Figure 2:**
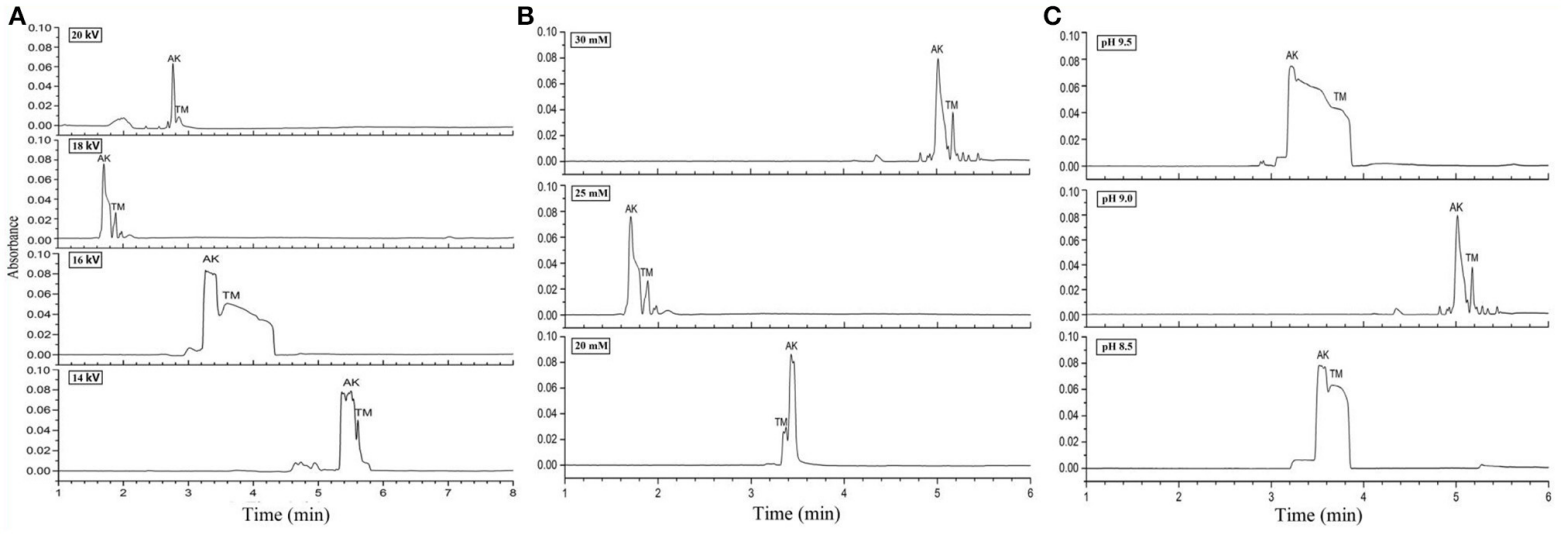
Electropherograms for mix standards of AK and TM. **(A)** 25 mM borate-borax with pH of running buffer at 9.2 under different separation voltages. **(B)** 18 kV separation voltage and pH of running buffer at 9.2 under different concentrations of borate-borax. **(C)** 25 mM borate-borax with 18 kV separation voltage at different pH values of running buffer.

**Figure 3:**
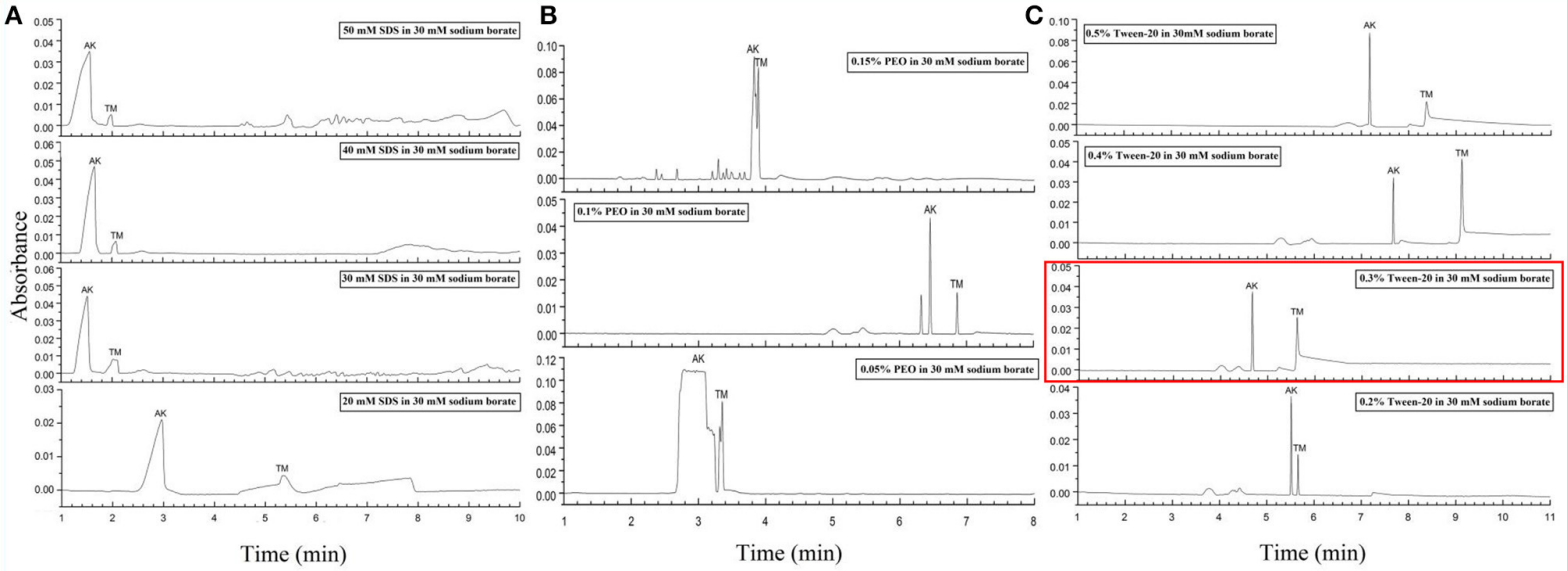
Electropherograms for mix standards of AK and TM with different dynamic coating modifiers. **(A)** 20, 30, 40, and 50 mM SDS. **(B)** 0.05, 0.1, and 0.15% PEO. **(C)** 0.2, 0.3, 0.4, and 0.5% Tween-20. The electropherogram with the optimized conditions was marked in a red rectangle. Separation conditions: 30 Mm borate-borax pH 9.0, with an 18 kV separation voltage.

In order to develop the method using dynamic coating capillary electrophoresis (DCCE) with high stability and recovery, which can simultaneously analyze the two main allergens (TM and AK) of shellfish, two aspects were taken into consideration. First, we investigated three influence factors of CZE, including the separate voltage, concentration, and pH of running buffer (Figure 2). Then, three modifiers for dynamic coating capillary electrophoresis were investigated (Figure 3).

The properties of the running buffer significantly affect the electroendosmotic flow (EOF) and the separation selectivity of analytes analyzed by CZE. Borate-borax buffer, which can provide acceptable efficiency of separation, good peak shapes, and reduction of protein adsorption, was selected as the running buffer because both AK and TM were acidic proteins. In the first step, we optimized three crucial parameters for CZE, including the separation voltage, buffer concentration, and buffer pH. The separation voltage influences the intensity of the electric field as well as the resolution and migration times of AK and TM. Different voltages from 14 to 20 kV with 25 mM borate-borax running buffer were tested. The results showed that migration time gradually decreased and peak efficiency increased with an increase from 14 to 18 kV (Figure 2A). However, in contrast with the voltage at 18 kV, higher levels (20 kV) unexpectedly led to poor separation, a longer migration time, an unstable baseline and low peak intensity.

Next, various concentrations of borate-borax running buffer at a voltage of 18 kV were examined (Figure 2B). Higher concentrations of running buffer usually shortened the separation time and improved resolution. Although we found that the migration times of both AK and TM were the shortest at a concentration of 25 mM of borate-borax running buffer, two sharp peaks were observed at a concentration of 30 mM with a migration time within 10 min. On the basis of the results of this study, an optimum borate-borax running buffer concentration of 30 mM was selected, as this electrolyte showed optimal resolution with an acceptable migration time.

Indeed, the pH value of the running buffer can affect the zeta-potential and the charge of analytes, further affecting separation efficiency and electrophoretic resolution. Then, we demonstrated the impact of the pH of the running buffer on the separation efficiency of AK and TM (Figure 2C). As shown in Figure 2C, two sharp peaks were observed at pH 9.0 for the borate-borax buffer, indicating the relatively high degree of separation of AK and TM obtained under these conditions. Collectively, we decided to employ a concentration of 30 mM and a pH of 9.0 of borate-borax running buffer as well as a voltage of 18 kV as the optimal parameters for AK and TM separation.

Despite the above interesting results in the separation of AK and TM due to the optimized conditions, the major issue was that the migration times of two peaks were still extremely close, which may lead to a reduction in practicability in fresh samples. To our knowledge, previous reports defined CZE conditions for protein separation as always involving the addition of poly(ethylene oxide) (PEO), SDS and Tween-20 (And and Chang, 2000; Esterman et al., 2016). PEO with high sieving ability has been particularly useful for concentrating negatively charged proteins and increasing the viscosity of the polymer solution (Chiu et al., 2003). SDS, an anionic surfactant, behaves as a modifier for dynamic coating to increase negative charge, increase the electrophoretic mobility of proteins that optimize resolution and speed, and decrease Coulombic interactions to minimize protein adsorption on the capillary wall (Tseng et al., 2002). In addition, dynamic coating by Tween-20, which can form complexes with proteins, is also considered able to change the effective electrophoretic mobility and prevent analyte adsorption on the capillary wall (Liu et al., 2010).

Thus, the second step of our condition optimization concerned the use of these modifiers for dynamic coating to improve electrophoretic mobility differences. Various concentrations of SDS solution ranging from 10 to 50 mM have been tested as dynamic coating modifier (Figure 3A and Figure S3). As presented in Figure 3A, an obvious decrease in migration time and increase in peak efficiency can be observed with the increase between 20 mM and the other SDS concentrations. Remarkably, at concentrations of 30 and 40 mM, two sharp peaks with a stable baseline were obtained. In addition, three PEO concentrations from 0.05 to 0.15% were also examined to modify CZE separation (Figure 3B). It is obvious that the addition of 0.1% PEO to the running buffer was essential for high separation efficiency and provided a sharpening of the protein peaks, but this process prolonged the migration time. Furthermore, the impact of five different concentrations from 0.1 to 0.5% of Tween-20 coating with running buffer were investigated (Figure 3C and Figure S3). Consequently, the improvement in resolution and reduction in migration time for both AK and TM were achieved at higher concentrations of Tween-20, especially at 0.3% Tween-20. Taken together, we selected 30 mM borate-borax (pH 9.0) with 0.3% (v/v) Tween-20 as a dynamic coating modifier for the separation of AK and TM at the voltage of 18 kV in terms of the peak shape, migration time, separation efficiency and electrophoretic resolution.

### Validation of IEC-DCCE and analysis of fresh shellfish samples

On the basis of these optimal conditions, the established IEC-DCCE method has been validated in terms of linear range, limit of detection (LOD) and limit of quantification (LOQ) using standards of AK and TM. Additionally, fresh samples of shrimp (*L. vannamei*) were treated to investigate precision (intra and inter-day relative standard deviation, RSD) and accuracy (recovery) of IEC-DCCE.

**Figure 4:**
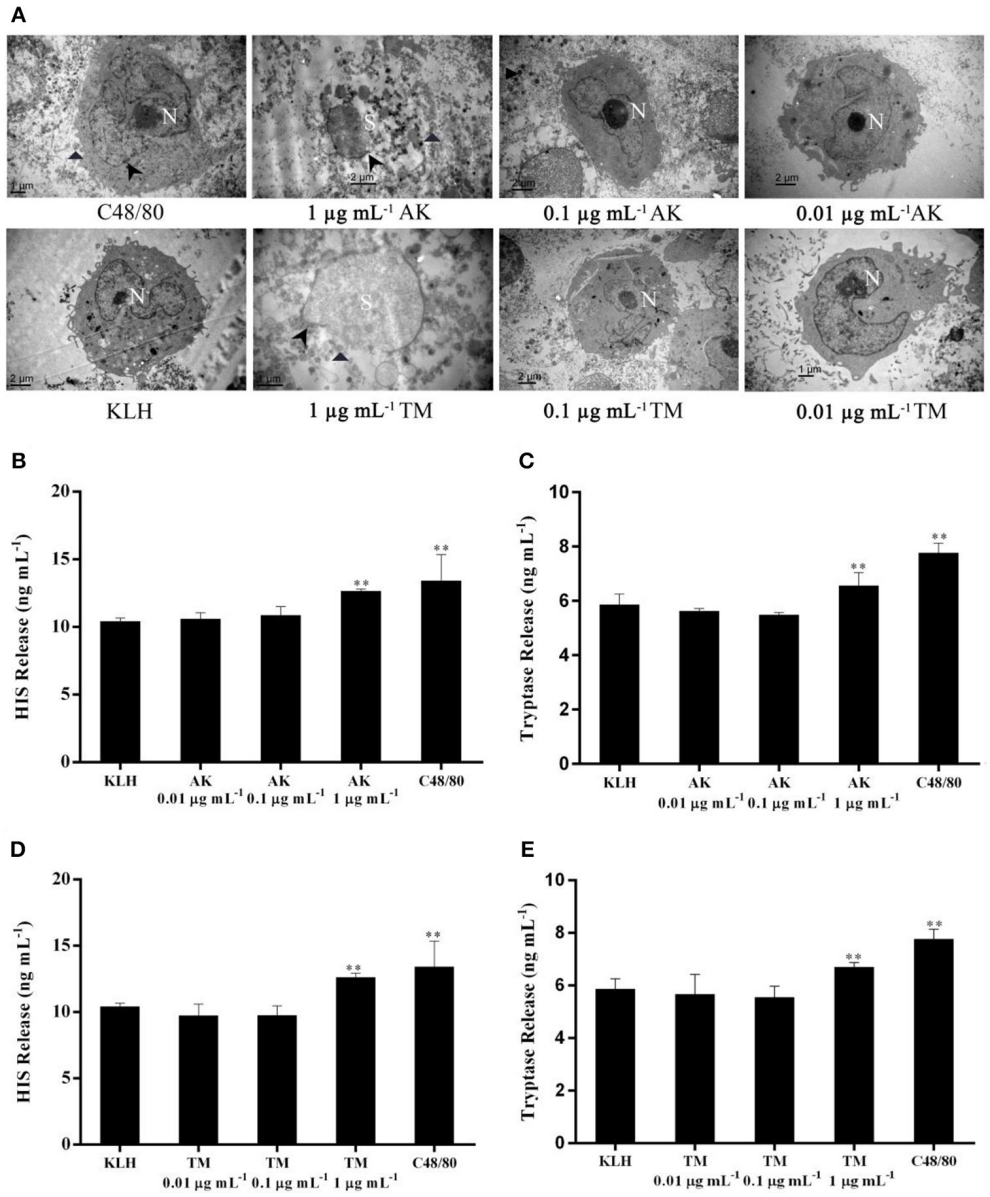
The effect of different concentrations at 0.01, 0.1 and 1 μg mL-1 of AK or TM on the degranulation of human mast cells. **(A)** TEM micrographs of LAD2 cells either unstimulated or stimulated using different concentrations of AK and TM. Non-segmented nucleus (N), segmented nucleus (S) and disrupted membranes (arrowheads) were observed. Parts of degranulated granules located outside the mast cell cytoplasm were also indicated (triangles). **(B,C)** show the levels of histamine and tryptase induced by different concentrations of AK (*n* = 3). **(D,E)** show the levels of histamine and tryptase induced by different concentrations of TM (*n* = 3). **p < 0.001 compared with negative control.

Five concentrations of standard mixture solutions (5, 10, 25, 40, 50 μg mL^−1^) were tested to determine the linear range of this method. The peak area linearly increased as the concentrations of AK and TM ranged from 5 to 50 μg mL^−1^ (Figure S4A). We also determined that the LODs were 1.2 μg mL^−1^ for AK and 1.1 μg mL^−1^ for TM (S/N = 3), respectively, and the LOQs were 4.0 μg mL^−1^ for AK and 3.7 μg mL^−1^ for TM (S/N = 10), respectively (Table 1). Compared to previous reports, the obtained LOD of IEC-DCCE was, to some extent, higher than those of ELISA (0.09 ng mL^−1^; Zhang et al., 2014) and real-time PCR (Eischeid et al., 2013). However, both the ELISA and real-time PCR methods have limitations in their application in quantifying allergens, as we indicated. Apparently, our newly developed IEC-DCCE has key advantages, which are low cost, simplicity and accurate detection of AK and TM simultaneously in 10 min without identification of gene sequences. Since the concentrations of AK and TM are relatively high in fresh shellfish, this IEC-DCCE is more suitable for determining major allergens in actual specimens. Moreover, only when the concentrations of both AK and TM are above the LODs we determined, these allergenic proteins could induce effective allergic response in human mast cell (LAD2) degranulation (Figure 4) as we discuss in detail in the following section. Generally, the threshold of food allergen is 10 μg of allergen protein/g (or mL) of food taken by individuals according to the Japanese Food Allergen Labeling Regulation (Shoji et al., 2017). Besides, another study indicated that intake of 2.5 g of shrimp protein could cause objective eliciting reactions in 10% of 436 allergic patients during oral food challenge (Ballmerweber et al., 2015).

**Table 1:** Validation of IEC-DCCE.

**Allergen**	**Migration time (min)**	**Inter-day RSD (*****n*** = **6)**		**Intra-day RSD (*****n*** = **6)**		**Linear equation**	**LOD (μg mL^−1^)**	**LOQ (μg mL^−1^)**
		**Migration time (%)**	**Peak area (%)**	**Migration time (%)**	**Peak area (%)**			
AK	4.1	6.9	4.7	6.8	6.4	y = 0.1x+0.07 *R*^2^ = 0.9968	1.2	4.0
TM	5.2	6.3	5.7	7.6	4.8	y = 0.1x−0.12 *R*^2^ = 0.9935	1.1	3.7

Next, we investigated the inter-day RSD for peak area and peak migration time bycontinuously testing one fresh samples of shrimp (*L. vannamei*) for six times, and the intra-day RSD were investigated by six fresh samples of shrimp (*L. vannamei*). The results showed that the established IEC-DCCE is reliable (Table 1 and Figure S4B). The recovery test was performed by randomly selecting five shrimp samples mixed with standards of AK and TM at a concentration of 5 μg mL^−1^, respectively. The recovery of AK ranged from 91.5 to 106.1%, while that of TM ranged from 94.0 to 109.5% (Tables S1, S2), indicating the accuracy of IEC-DCCE.

To test the ability of this IEC-DCCE to function effectively and applicably in the presence of AK and TM, we analyzed 10 different species of fresh shellfish samples from a local market. The concentrations of the two allergens in shellfish samples were back-calculated from the calibration curves. The final amounts of AK and TM existed in the fresh shellfish samples were indicated as the mass of allergens/total mass of shellfish powder, which was calculated through the concentrations of the two allergens in shellfish samples. The results are summarized in Figure S5 and Table 2. These data showed that AK and TM could be detected in almost all of the shellfish species we collected with abundances between 0.97 and 6.35 mg per g shellfish powder. Interestingly, the content of TM in fresh shellfish obtained by ELISA (Lin et al., 2018) were higher than we tested here, while the content of TM in cooked shellfish were close, which may due to the discrepancy between two methods. Notably, a high concentration of AK was detected in *E. carinicauda*, but TM was not detected in concentrations above the LOD in *P. trituberculatus*. Our analysis of fresh samples provides information about the amounts of AK and TM in 10 main shellfish species that are frequently consumed and highlights the possibility of the newly developed IEC-DCCE for detecting the two major allergens in shellfish, which may help the industrial and government allergen-labeling of shellfish and shellfish products.

**Table 2:** Amounts of AK and TM in fresh shellfish samples detected by IEC-DCCE.

**Shellfish species**	**Arginine kinase**		**Tropomyosin**	
	**Measured Conc. (μg mL^−1^) ± SD^a^**	**(mg/g) ± SD^a^**	**Measured Conc. (μg mL^−1^) ± SD^a^**	**(mg/g) ± SD^a^**
*Litopenaeus vannamei*	9.80 ± 1.80	1.96 ± 0.36	10.00 ± 0.25	2.00 ± 0.050
*Fenneropenaeus chinensis*	13.10 ± 1.85	2.62 ± 0.37	12.70 ± 1.40	2.54 ± 0.28
*Trachypenaeus curvirostris*	11.60 ± 1.40	2.32 ± 0.28	15.75 ± 0.90	3.15 ± 0.18
*Penaeus monodon*	10.65 ± 1.40	2.13 ± 0.28	15.15 ± 1.40	3.03 ± 0.28
*Macrobrachium nipponense*	7.65 ± 0.85	1.53 ± 0.17	6.00 ± 0.35	1.2 ± 0.069
*Procambarus clarkii*	7.70 ± 0.75	1.54 ± 0.15	11.9 ± 1.15	2.38 ± 0.23
*Metapenaeus ensis*	11.25 ± 1.55	2.25 ± 0.31	4.85 ± 0.31	0.97 ± 0.061
*Exopalaemon carinicauda*	31.75 ± 0.75	6.35 ± 0.15	10.65 ± 2.00	2.13 ± 0.40
*Eriocheir sinensis*	12.75 ± 0.85	2.55 ± 0.17	5.00 ± 0.21	1.00 ± 0.042
*Portunus trituberculatus*	18.75 ± 1.55	3.75 ± 0.31	ND^a^	ND^a^

a *n = 3; ND^a^, not detected above the LOD*.

### Biological significance of the LOD

Theoretically, the LOD, a threshold concentration in the validation of analytical methods, is considered best when lowest for analysis of hazardous substances polluted in food, especially analytes such as pathogens (Wang et al., 2015), veterinary drugs (Wu et al., 2016) and heavy metals (Akinyele and Shokunbi, 2015). Nevertheless, as two native functional proteins existed in aquatic animals, AK and TM actually would not induce adverse reactions in susceptible individuals when under the threshold amount. Thus, we consider that it is necessary to have fast, effective and feasible analytical techniques that can also reflect the biological significance of the LOD in allergen detection.

Mast cells play a central role in allergic and inflammatory disorders by inducing degranulation and inflammatory mediator release, so the mast cell degranulation assay is usually employed to measure the allergenicity of potential allergens (Wang et al., 2015). To test whether a series of concentrations of AK and TM above or below the LOD could induce human mast cell LAD2 degranulation, we investigated levels of two biomarkers (histamine and tryptase) and morphological changes of LAD2 cells. The C48/80-stimulated group was used as the positive control because C48/80 is a potent activators to increase intracellular Ca^2+^ concentration in mast cells, leading to degranulation of proinflammatory mediators that can further induce the release of histamine and lipid mediators (Pundir et al., 2014).

We found that AK or TM at a concentration of 1 μg mL^−1^ was capable of stimulating LAD2 cell degranulation assessed by morphological changes and the release of two biomarkers (Figure 4). As shown in Figure 4A, C48/80-stimulated LAD2 cells showed an irregular nucleus and secretory granules distributed outside the cytoplasm, exhibiting a loss of membrane density. Meanwhile, LAD2 cells stimulated by KLH (as negative control) displayed a non-segmented nucleus, narrow surface folds and a cytoplasm with secretory granules. Remarkably, LAD2 cells treated with AK (1 μg mL^−1^) or TM (1 μg mL^−1^) showed completely segmented nuclei, disruption of the cell membrane, numerous secretory granules and some partly empty granules out of the membrane. However, LAD2 cells treated with AK or TM at both 0.1 and 0.01 μg mL^−1^ presented a normal ultrastructure similar to that of KLH-stimulated cells, indicating that no degranulation occurred when LAD2 cells were challenged at these two concentrations.

In addition, the effects of three concentrations of AK and TM on the release of histamine (Figures 4B,D) and tryptase (Figures 4C,E) from LAD2 mast cells were also investigated. Incubation of mast cells with 1 μg mL^−1^ AK, 1 μg mL^−1^ TM or C48/80 significantly (*p* < 0.001) increased the levels of histamine and tryptase compared with the KLH group. In contrast, AK and TM at concentrations of 0.1 and 0.01 μg mL^−1^ did not activate LAD2 cell degranulation and chemokine production, consistent with the phenomenon observed by TEM. Altogether, our IEC-DCCE method was able to simply, effectively, and simultaneously detect AK and TM in shellfish at an allergenic concentration, suggesting the biological significance of the LOD.

## Conclusion

This paper demonstrates a simple, improved CE method for determination of two major allergens in shellfish that have very close molecular weights and pI (isoelectric point) values and are both acidic glycoproteins. The protocol presented here is the first report of the simultaneous separation of AK and TM by IEC-DCCE. To date, this method is the most effective and applicable method available for accurately and quantitatively detecting allergens. Additionally, only when the concentrations of AK and TM are above the limits of detection reported here are these allergenic proteins able to induce human mast cell LAD2 degranulation. Hence, our method also reflects the biological significance of the LOD in natural functional protein detection.

By using 30 mM borate-borax, pH 9.0, with 0.3% (v/v) Tween-20 as the dynamic coating modifier for the separation, the migration time, separation efficiency and electrophoretic resolution greatly improved. With good accuracy and precision, the method can overcome the problem of cross-reactivity associated with immunoassays. Further work is underway to develop a multiple-CE detection method for allergens in seafood.

In addition to the establishment of the IEC-DCCE platform, an important finding from this study was that fresh shellfish samples from different species and containing diverse protein components can be accurately analyzed for the amounts of AK and TM with simple sample preparation. With this method, the presence of the two major allergens was detected in 10 shellfish species.

These data clearly illustrate how the IEC-DCCE method described here could be used to quantify the two major allergens, AK and TM, in shellfish. These results also highlight the applicability of CE-based methods for the simultaneous detection of diverse allergens at the allergenic concentration range in complicated food samples.

## Author contributions

LF and YW designed research. JZ and CW analyzed data. LF, JZ, and CW performed research. LF, JZ, and YW wrote the paper. XL contributed new reagents or analytic tools. LZ developed software necessary to perform and record experiments.

### Conflict of interest statement

The authors declare that the research was conducted in the absence of any commercial or financial relationships that could be construed as a potential conflict of interest.
