# Supplementary material for: Ion-Exchange Chromatography Coupled With Dynamic Coating Capillary Electrophoresis for Simultaneous Determination of Tropomyosin and Arginine Kinase in Shellfish
Source: Front Chem. 2018 Jul 25;6:305. doi: 10.3389/fchem.2018.00305 (PMC6068269; doi:10.3389/fchem.2018.00305)
Supplement: Supplementary file 1 [file Data_Sheet_1.docx]

***Supplementary Material***

**Ion-Exchange Chromatography Coupled with Dynamic Coating Capillary Electrophoresis for Simultaneous Determination of Two Major Allergens in Shellfish**

Linglin Fu^1,2^, Jinru Zhou^1,2^, Chong Wang^1^, Xiaohui Li^1^, Lei Zheng^3^, Yanbo Wang^1,2,*^

^1^Food Safety Key Laboratory of Zhejiang Province, School of Food Science and Biotechnology, Zhejiang Gongshang University, Hangzhou 310018, China

^2^Zhejiang Engineering Institute of Food Quality and Safety, Zhejiang Gongshang University, Hangzhou 310018, China

^3^School of Food Science and Engineering, Hefei University of Technology, Hefei 230009, China

*** Corresponding author**

Address: 18 Xue Zheng Street, Hangzhou, 310018, China

Tel.: +86-571-28008963

Email: wyb1225@163.com (Dr. Y. Wang)

# Figure S1. SDS-PAGE analysis of shrimp protein crude extract and IEC treated protein.
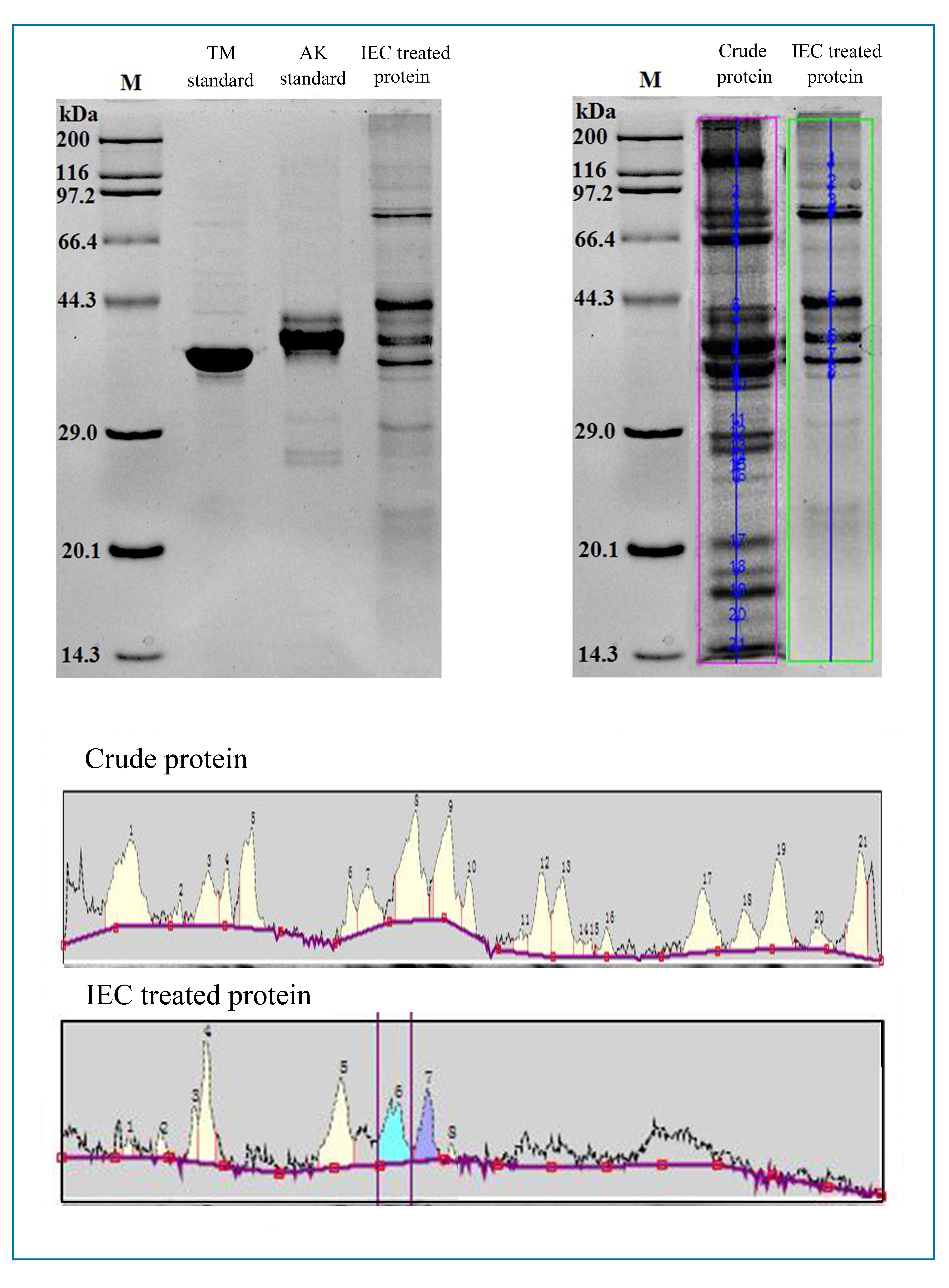


# Figure S2. Elution characteristics and SDS-PAGE analysis of shrimp protein crude extract. 3 mL of shrimp crude extract was loaded into a DEAE Sepharose F.F. column at pH 7.5 gradient elution using 0.1~0.5 M NaCl as the eluent. Effluents were collected every 5 minutes and monitored using NanoDrop 2000 at 280 nm. S, shrimp crude extract; M, molecular weight markers. Three peaks represent maximal absorbance of protein in effluents and were analyzed by 12% reduced SDS-PAGE.


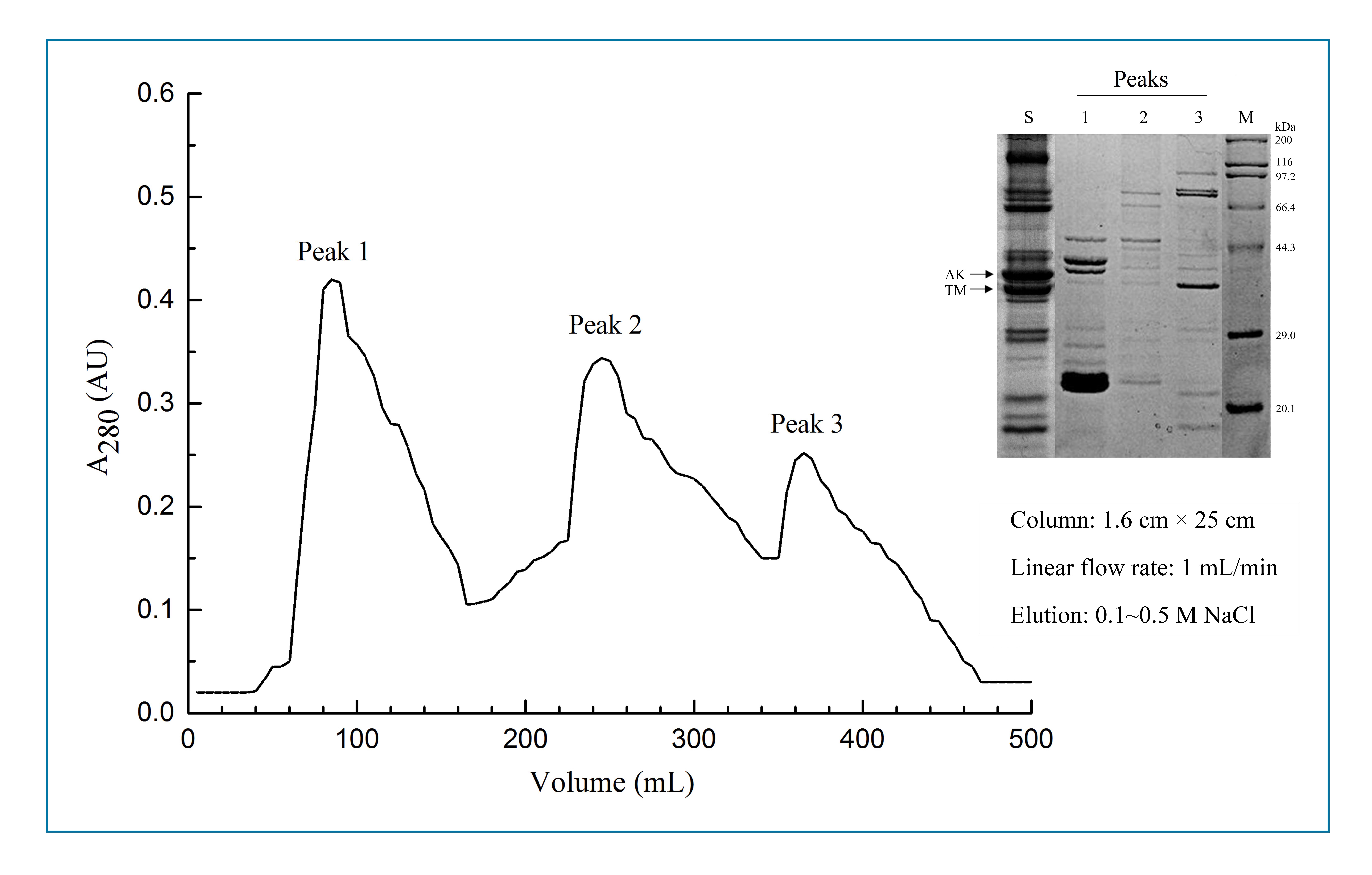


**Figure S3.** **Electropherograms showing the effect of coating modifiers on the separation efficiency of AK and TM.** (A) Without modifier. (B) 0.1% (v/v) Tween-20. (C) 10 mM SDS. The other conditions were the same as Figure 3.





**Figure S4. Validation of IEC-DCCE.** (A) Calibration curves of different concentrations of AK and TM vs peak area. (B) Electropherograms of six fresh samples of shrimp (*Litopenaeus vannamei*).


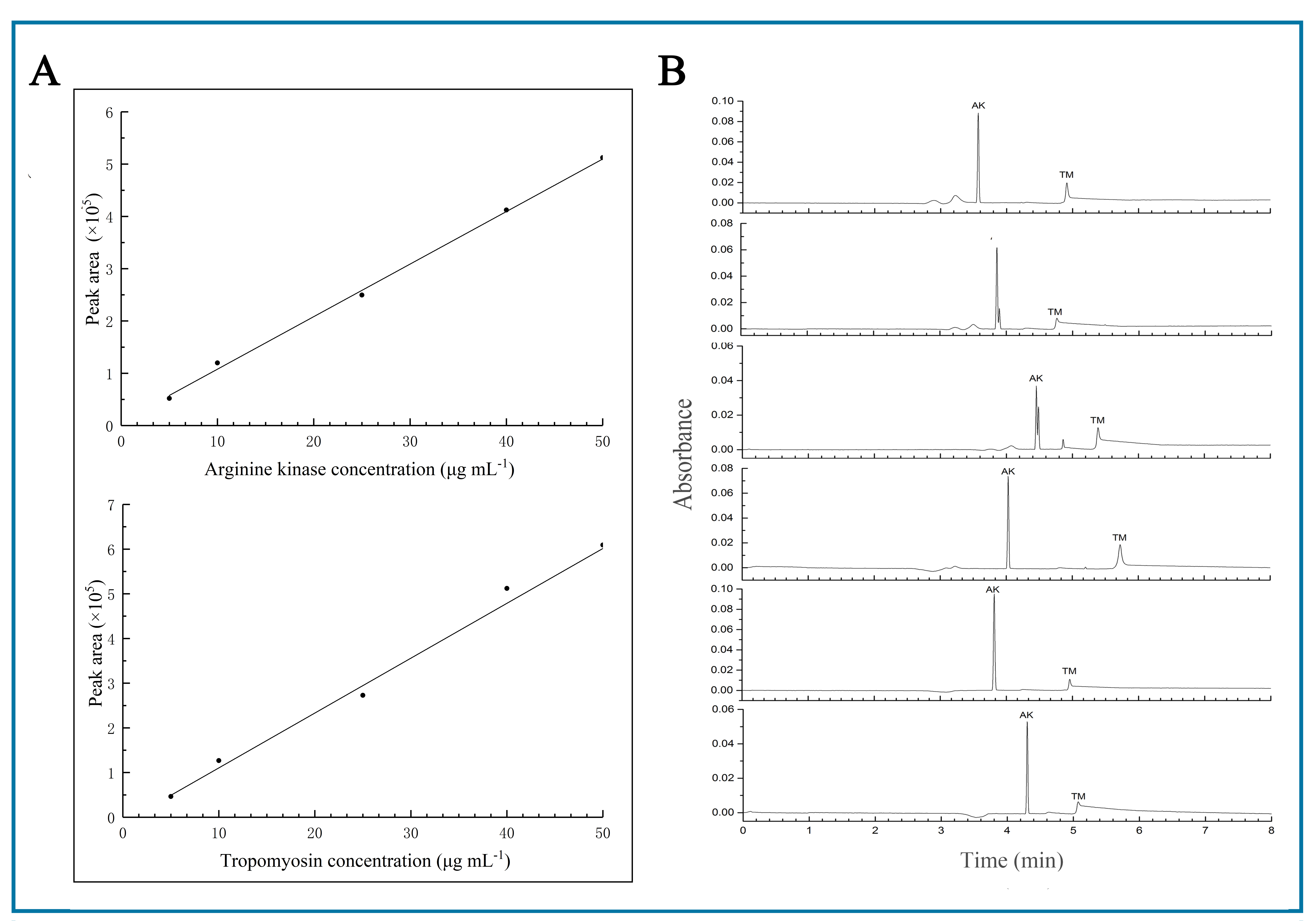


**Figure S5.** **Electropherograms of the AK and TM analysis for 10 different species of shellfish.** Separation conditions: 30 Mm borate-borax pH 9.0, coating 0.3% (v/v) Tween-20, with an 18 kV separation voltage.


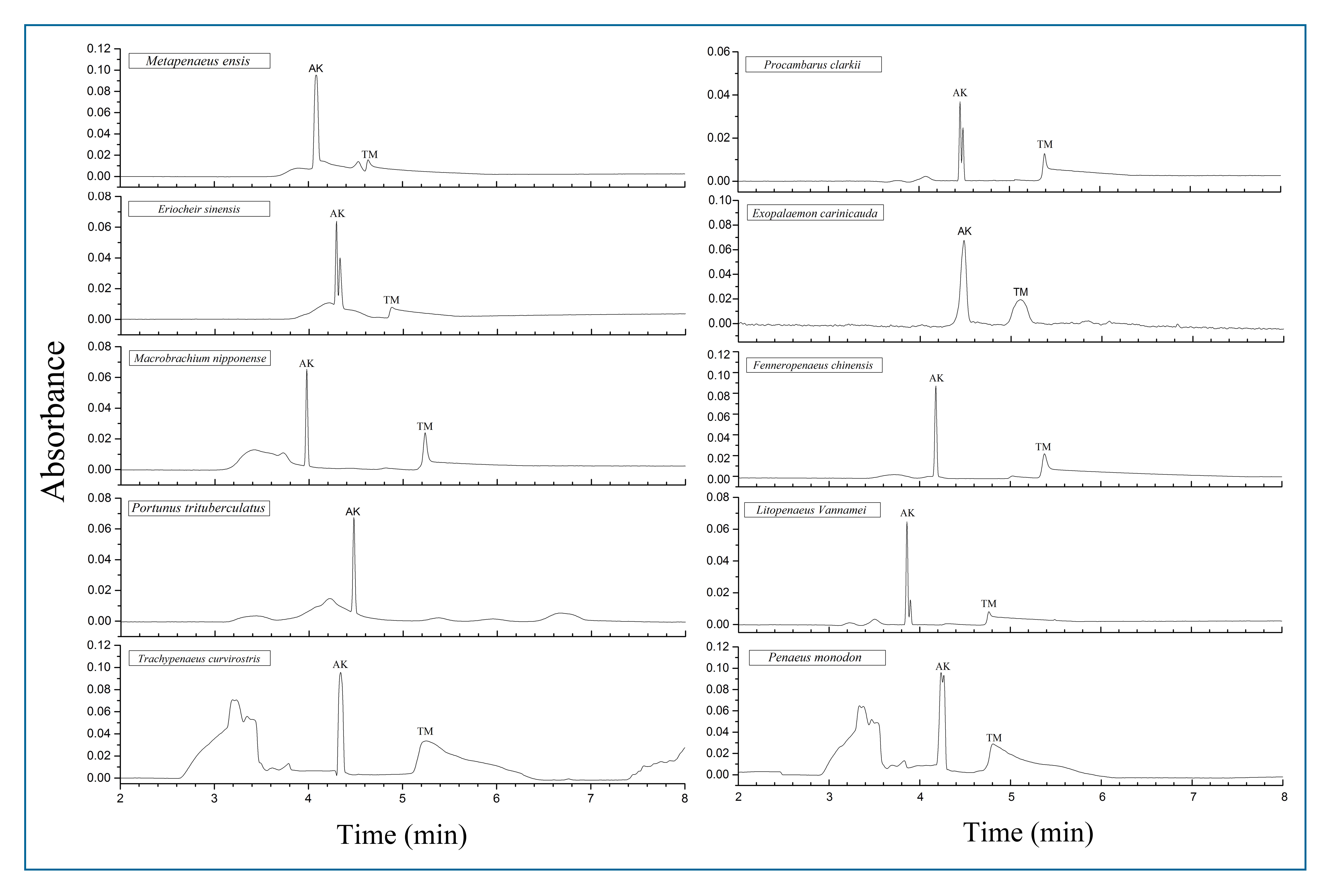


**Table S1 The recovery of AK by IEC-DCCE.**

| **Sample No.** | **Natural content of AK (μg mL^-1^)** | **Spiked (μg mL^-1^)** | **Measured (μg mL^-1^)** | **Recovery (%)** |
| --- | --- | --- | --- | --- |
| S1 | 11.3 | 16.3 | 17.3 | 106.1 |
| S2 | 12.3 | 17.3 | 18.3 | 105.7 |
| S3 | 10.3 | 15.3 | 14 | 91.5 |
| S4 | 15.3 | 20.3 | 21.3 | 104.9 |
| S5 | 14.3 | 19.3 | 20.3 | 105.1 |

**Table S2 The recovery of TM by IEC-DCCE.**

| **Sample No.** | **Natural content of TM (μg mL^-1^)** | **Spiked (μg mL^-1^)** | **Measured (μg mL^-1^)** | **Recovery (%)** |
| --- | --- | --- | --- | --- |
| S1 | 10.8 | 15.8 | 15.2 | 96.2 |
| S2 | 10.7 | 15.7 | 17.2 | 109.5 |
| S3 | 11.2 | 16.2 | 16.2 | 100 |
| S4 | 10.1 | 15.1 | 14.2 | 94 |
| S5 | 10.2 | 15.2 | 16.4 | 107.8 |
